# High-Mobility Tellurium Thin-Film Transistor: Oxygen Scavenger Effect Induced by a Metal-Capping Layer

**DOI:** 10.3390/nano15060418

**Published:** 2025-03-08

**Authors:** Seung-Min Lee, Seong Cheol Jang, Ji-Min Park, Jaewon Park, Nayoung Choi, Kwun-Bum Chung, Jung Woo Lee, Hyun-Suk Kim

**Affiliations:** 1 Department of Materials Science and Engineering, Chungnam National University, Daejeon 34134, Republic of Korea; 2 Department of Energy and Materials Engineering, Dongguk University, Seoul 04620, Republic of Korea; 3 Department of Physics, Dongguk University, Seoul 04620, Republic of Korea; 4 School of Materials Science and Engineering, Pusan National University, Busan 46241, Republic of Korea

**Keywords:** p-type semiconductor, tellurium, high mobility, metal-capping layer, thin-film transistors

## Abstract

With the ongoing development of electronic devices, there is an increasing demand for new semiconductors beyond traditional silicon. A key element in electronic circuits, complementary metal-oxide semiconductor (CMOS), utilizes both n-type and p-type semiconductors. While the advancements in n-type semiconductors have been substantial, the development of high-mobility p-type semiconductors has lagged behind. Recently, tellurium (Te) has been recognized as a promising candidate due to its superior electrical properties and the capability for large-area deposition via vacuum processes. In this work, an innovative approach involving the addition of a metal-capping layer onto Te thin-film transistors (TFTs) is proposed, which significantly enhances their electrical characteristics. In particular, the application of an indium (In) metal-capping layer has led to a dramatic increase in the field-effect mobility of Te TFTs from 2.68 to 33.54 cm^2^/Vs. This improvement is primarily due to the oxygen scavenger effect, which effectively minimizes oxidation and eliminates oxygen from the Te layer, resulting in the production of high-quality Te thin films. This progress in high-mobility p-type semiconductors is promising for the advancement of high-performance electronic devices in various applications and industries.

## 1. Introduction

Thin-film transistors (TFTs) are crucial components not only in cutting-edge displays but also in a range of prospective electronic devices, such as flexible/stretchable electronics and monolithic three-dimensional (3D) complementary metal oxide semiconductors (CMOS) [1,2,3,4,5]. The deployment of both n- and p-type semiconductors is essential for developing high-performance circuits in future electronics. Recently, amorphous InGaZnO (a-IGZO) has garnered significant attention due to its superior qualities relative to Si-based semiconductors, including high mobility, excellent transparency owing to its large bandgap, extensive area uniformity, and low leakage current that collectively contribute to decreased power consumption [6,7,8,9]. In p-type oxide semiconductors, hole movement predominantly occurs within the valence band, characterized by strongly localized oxygen 2p orbitals [10,11,12,13]. Additionally, the generation of holes is hindered by the high formation energy associated with the intrinsic acceptor, which leads to substantial effective hole mass and diminished carrier mobility. Consequently, the development of new high-performance p-channel materials, distinct from oxides, is imperative for diversifying electronic applications.

Numerous high-mobility p-type transistors utilizing two-dimensional materials have been developed; however, these materials often necessitate elevated growth temperatures to achieve optimal film quality [14,15,16,17]. Furthermore, issues regarding large-area fabrication persist, influenced by numerous factors such as growth substrates, conditions, and uniformity control. Recently, tellurium (Te), a group VI quasi-2D material, has emerged as a favorable p-type semiconductor, offering advantages such as piezoelectric, photoconductive, and thermoelectric properties alongside high hole mobility [18,19,20,21,22]. The isotropic structure of Te, characterized by Te atoms covalently bonded to two adjacent atoms forming a triangular helix chain along the [0001] direction in a hexagonal arrangement via van der Waals forces, supports the formation of a naturally passivated surface without dangling bonds—critical for achieving clean, high-performance interfaces. Predominantly, Te is fabricated through evaporation-based deposition at cryogenic temperatures, molecular beam epitaxy, and liquid processes, which are unsuitable for large-area mass production [23,24,25]. Despite the advancements in room-temperature, large-area-capable sputtering processes for Te TFTs, there remains a trade-off between mobility and the on/off ratio [23,26,27]. Recent studies have explored Selenium-Te alloy p-type TFTs utilizing thermal evaporation or pulsed laser deposition (PLD); however, their mobility has yet to meet expectations [28,29].

Significant research efforts have focused on improving mobility through structural modifications, such as the inclusion of bilayers or metal-capping layers [30,31,32]. The metal-capping layer, in particular, is a straightforward technique that significantly enhances the mobility of transistors by adding a metal layer atop semiconductors. This method proves versatile, effectively influencing both p-type and n-type semiconductors, although the mechanism of mobility enhancement varies with the specific semiconductor and metal layer combinations. This study focuses on improving the mobility of the TFTs, which were fabricated using a sputtering process at room temperature, by incorporating a metal-capping layer. After optimizing the characteristics of Te, an indium (In) metal-capping layer was applied to the Te TFTs to boost mobility. The application of an In-capping layer notably enhances mobility by approximately 12 times without compromising the on/off ratio. Moreover, the bias stability was unaffected by the introduction of the capping layer.

## 2. Materials and Methods

**Figure 1:**
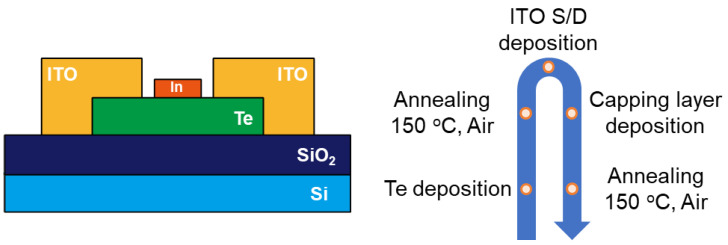
Schematic diagram of cross-sectional In-capped Te TFT and fabrication procedure.

Te TFTs were fabricated on heavily doped p-type Si substrates with a 100-nanometer-thick thermally grown SiO_2_ dielectric layer. The Te-active layers were deposited by a sputtering system (I.T.S, Ansan-si, Republic of Korea) at room temperature using a Te target (99.99%, Toshima, Tokyo, Japan) with RF powers set to 20 W. The working pressure was maintained at 10 mTorr, while the Ar gas flow rate was consistently kept at 10 sccm. Following the deposition of the active layers, thermal annealing was carried out at 150 °C for 1 h in an air ambient using rapid thermal annealing (RTA, Han TECH, Ulsan, Republic of Korea). Source-drain electrodes were then formed by depositing a 100-nanometer-thick ITO film using DC sputtering (EI Technology, Busan, Republic of Korea), resulting in a channel with a width (W) and length (L) of 800 and 200 μm, respectively. Subsequently, 2.5-nanometer-thick indium (In) metal layers were deposited between the source/drain electrodes with dimensions of W/L = 1300/100 μm using the sputtering process (EI Technology, Korea). After the deposition of the capping layer, a second thermal annealing was conducted at 150 °C for 1 hr in an air ambient by RTA. All layers were defined using a shadow mask. Figure 1 shows the schematic diagram of the cross-sectional In-capped Te TFT and the fabrication procedure. All TFTs were characterized at RT (approximately 25 °C) in dark and ambient conditions. The electrical properties were measured using a Keithley 4200-SCS (Tektronix, Beaverton, OR, USA).

For the crystallinity of Te thin film before and after the annealing process, XRD was measured by a D2 Phaser instrument (Bruker, Billerica, MA, USA) using Te films with a thickness of 50 nm. Except for XRD, all samples used for characterization were set to 7 nm for Te and 2.5 nm for the In-capping layer. To see the chemical structure of Te, XPS (Versaprobe II, ULVAC-PHI, Chigasaki, Japan) was measured using a monochromatic Al Kα (hν = 1486.7 eV). UV–vis was measured by SCINCO, S-3100 (SCINCO, Seoul, Republic of Korea), to see the optical properties such as transmittance and optical bandgap.

## 3. Results and Discussion

**Figure 2:**
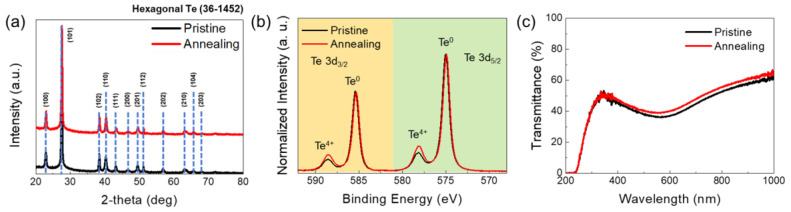
(**a**) X-ray diffraction (XRD) patterns. (**b**) Te 3d XPS spectra. (**c**) Optical transmittance of the pristine and annealed Te films at 150 °C.

Figure 2a displays the X-ray diffraction (XRD) spectra of Te thin films annealed at a temperature of 150 °C. The XRD spectrum includes a total of 12 distinct peaks located at 23.04°, 27.6°, 38.5°, 40.4°, 43.3°, 46.8°, 49.6°, 51.3°, 56.9°, 63.1°, 65.6 °, and 67.9°, corresponding to the crystallographic planes (100), (101), (102), (110), (111), (200), (201), (112), (202), (210), (104), and (203), respectively. The Te thin films exhibit similar polycrystalline phases regardless of annealing, with the main peak at (101) and an XRD refraction pattern reminiscent of hexagonal Te as previously reported (JCPDS Card No. 36-1452). These findings confirm that physical crystallinity changes due to annealing up to 150 °C are minimal. Above 150 °C, volatilization occurs, causing the film edges to blur and its thickness to gradually decrease, as illustrated in Figure S1.

X-ray photoelectron spectroscopy (XPS) analysis was utilized to explore the chemical structure of the Te thin films. Figure 2b presents the Te 3d XPS spectra of Te thin films as a function of annealing, featuring four peaks with binding energies around 588.56, 585.44, 578.17, and 574.94 eV. The peaks near 588.56 and 585.71 eV correspond to the Te 3d_3/2_, and the peaks near 578.17 and 574.94 eV correspond to Te 3d_5/2_ [26,29,33,34]. The peaks around 588.56 and 578.17 eV indicate the Te^4+^ state, which means oxidation states of Te, and the peaks around 585.44 and 574.94 eV indicate the Te^0^ state, representing the neutral metallic Te state. Both pristine and annealed Te thin films predominantly exhibit the neutral Te^0^ state rather than the oxidized Te^4+^ state. The peaks corresponding to Te^4+^ in both Te 3d_3/2_ and Te 3d_5/2_ are related to the oxidation state and increased with annealing, indicating oxidation at the annealing temperature of 150 °C.

UV/vis spectroscopy was utilized to explore the optical properties of Te thin films before and after annealing processes. As depicted in Figure 2c, the transmittance of the Te thin film escalated from an average of 39.9% to 42.5% in the visible ray region (380–700 nm) after annealing, corroborating the oxidation of the Te thin film suggested by XPS results.

**Figure 3:**
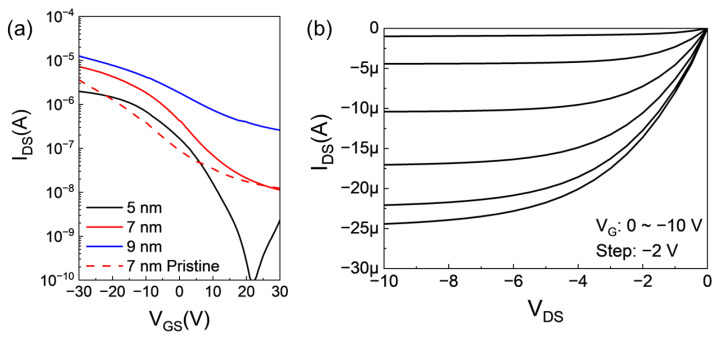
(**a**) Transfer curves of Te TFTs under various conditions, and (**b**) output curves of 7-nanometer-thick annealed Te TFTs at 150 °C.

Electrical characteristics of the p-type Te TFT were examined by measuring transfer curves corresponding to varying Te thickness, as illustrated in Figure 3a. Transfer curves were measured over a gate voltage range from 30 to −30 V at a drain voltage of −1 V. Table 1 summarized the representative transfer parameters of the Te TFTs in this work. A pristine 7-nanometer-thick Te TFT exhibited typical p-type behavior with a mobility, on/off ratio, and V_on_ value of 2.16 cm^2^/Vs, 2.95 × 10^2^, and −3.11 V, respectively. The annealing process improved the Te TFT electrical characteristics, changing the mobility, on/off ratio, and V_on_ to 2.68 cm^2^/Vs, 6.36 × 10^2^, and −0.88 V, respectively. It is known that the Te TFTs have an increased mobility and a decreased on/off ratio as their thickness increases. Conversely, as their thickness decreases, their mobility decreases, and the on/off ratio increases. Specifically, a 5-nanometer-thick Te TFT showcased a high on/off ratio of 2.24 × 10^4^, albeit with a comparatively low on current, resulting in diminished field-effect mobility of 0.58 cm^2^/Vs. Conversely, the 9-nanometer-thick Te TFT, the thickest sample assessed, evidenced a mobility of 3.92 cm^2^/Vs and an on/off ratio of 4.86 × 10. Output curves of the 7-nanometer-thick Te TFT in Figure 3b demonstrated conventional p-type transport behavior with ohmic-like contacts.

**Figure 4:**
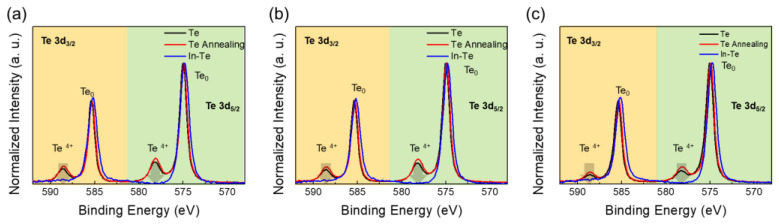
XPS Te 3d spectra of pristine Te, annealed Te at 150 °C, and In-capped annealed Te at 150 °C for (**a**) back, (**b**) bulk, and (**c**) front channels.

To enhance the Te TFT mobility, an indium (In) metal-capping layer was applied atop the Te thin film. Subsequent to the In deposition by sputtering process, an additional annealing at 150 °C for 1 h was performed. The XPS Te 3d analysis was conducted on three distinct regions of the Te thin film under different conditions: pristine Te, annealed Te, and annealed In-capped Te. Figure 4a–c show the XPS Te 3d spectra obtained from the back channel (interface between Te and In, or the surface of Te), bulk Te, and front channel of Te, respectively. Both pristine and annealed Te exhibited not only the metallic Te^0^ peak but also the oxidation peak of Te^4+^ in both Te 3d_3/2_ and Te 3d_5/2_ at the back channel, which is the surface of the Te film. There is no significant change in peaks across the back channel, bulk, and front channel, indicating that oxygen is uniformly distributed throughout the entire Te thin film. After the introduction of the In-capping layer, the oxidation peaks of Te^4+^ for both Te 3d_3/2_ and Te 3d_5/2_ decrease significantly and are almost undetectable. This phenomenon occurs not only in the back channel area but is also consistently observed in both the bulk and front channels.

**Figure 5:**
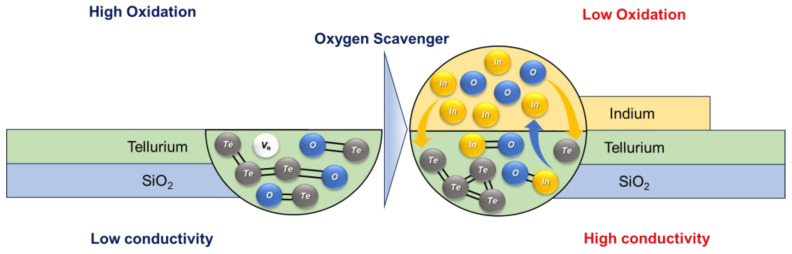
Schematic representation of the oxygen scavenger effect in In-capped Te.

According to XPS results, the oxidation peak of Te diminished following the introduction of an In-capping layer. Figure 5 illustrates a schematic of the mechanism occurring when a metal-capping layer of In is applied to Te. During the In sputtering process and subsequent post-annealing process, In and Te diffuse each other, and the oxygen bound to Te undergoes bond dissociation upon the introduction of In, subsequently combining with In atoms. This occurrence is attributed to the differences in Gibbs free energy of formation (ΔG_f_) between the In_2_O_3_ (−830.7 eV) and TeO_2_ (−318.0 eV), which indicate their oxidation tendencies [32,35,36]. A lower ΔG_f_ suggests a higher propensity to form oxide. Consequently, In scavenges oxygen from Te, forming In_2_O_3_ and reinforcing the Te network. Moreover, the effective diffusion of In into Te during the sputtering and post-annealing process facilitates the removal of oxygen from Te, thereby strengthening the Te network and enhancing current transport, which leads to improved mobility.

**Figure 6:**
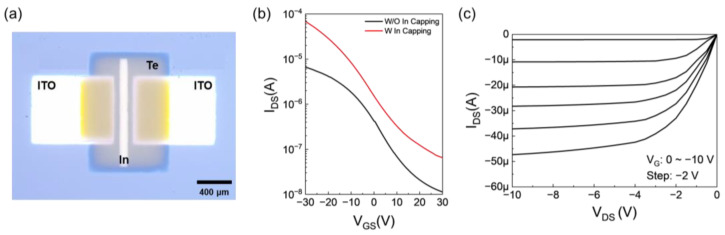
(**a**) Optical image of an In-capped Te TFT. (**b**)Transfer curves of an In-capped Te TFTs. (**c**) Output curves of an In-capped Te TFT.

A TFT was fabricated to validate the mobility enhancement achieved by the In-capping layer. Figure 6a shows the optical microscope (OM) image of In-capped Te TFTs. The In-capping layers, deposited between the source/drain electrodes with dimensions of W/L = 1300/100 μm by the sputtering process, are distinctly separated into islands and are not directly connected to the S/D electrode. Figure 6b represents the transfer curve of the Te TFTs enhanced by a In metal-capping layer. With the In-capping layer implemented, there has been a notable increase in current. Consequently, the mobility has improved 12 times, advancing from 2.68 to 33.54 cm^2^/Vs, and the on/off ratio has been enhanced to 1.05 × 10^3^. V_on_ showed a slight positive shift from −3.11 to −2.21 V. This enhancement is attributed to the disruption of the Te-O bond and the fortification of the Te-Te bond, facilitating smoother carrier transport, as verified by XPS. Figure 6c illustrates the output curves of the Te TFT with the In-capping layer, demonstrating ohmic-like contact and a higher current than the Te TFT without the layer. Table S1 shows a summary of the reported Te-related TFTs [23,24,26,28,29,37,38,39,40]. Various deposition methods have been introduced to fabricate Te thin films, such as cryogenic evaporation, sputtering, MBE, and PLD. Recently, studies have been reported to further improve the mobility of Te TFTs through Se alloying, encapsulation, and partial oxide. In this study, enhancement of Te mobility was demonstrated through an In-capping layer, which enabled the Te TFT to improve the mobility by approximately 10 times.

**Figure 7:**
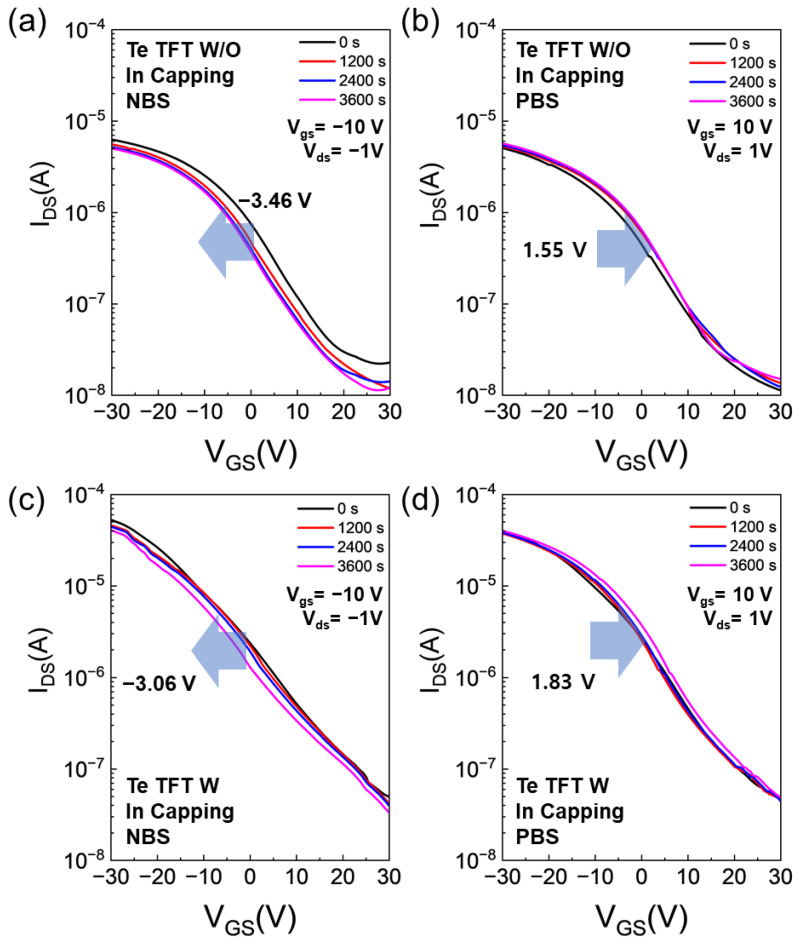
Transfer curves under bias stress stability test for Te TFTs (**a**,**b**) and In-capped Te TFTs (**c**,**d**), for NBS (**a**,**c**) and PBS (**b**,**d**).

Moreover, stability tests under both negative bias stress (NBS) and positive bias stress (PBS) were conducted to evaluate the performance of both annealed Te TFTs and In-capped Te TFTs. For NBS, the applied gate voltage (V_gs_) and drain voltage (V_ds_) were −10 V and 1 V, respectively. For PBS, V_gs_ and V_ds_ were set to 10 V and 1 V, respectively. The transfer curves for Te TFTs and In-capped Te TFTs are depicted in Figure 7. With increasing stress time, all the Te TFTs exhibit negative and positive shifts under NBS and PBS, respectively. No significant differences in bias stability were observed, even after capping. However, there are notable differences in the shifts of transfer curves under PBS and NBS. At negative bias and hole trapping, and at positive bias, electron trapping serves as the primary degradation mechanism. Consequently, trapping of relatively large amounts of holes occurs more frequently, resulting in poorer NBS stability compared to PBS.

## 4. Conclusions

This study demonstrates the significant mobility enhancement of the Te TFT employing a metal-capping layer. Te can be deposited via a sputtering process at room temperature, with its electrical properties modifiable through the control of thickness. The integration of an In metal layer markedly elevates the mobility from 2.68 to 33.54 cm^2^/Vs and simultaneously improves the on/off ratio. Analytical results from XPS suggest that the enhanced performance results from In diffusing into Te during the sputtering process, acting as an oxygen scavenger, and thereby disrupting the bonding between Te and oxygen to reinforce the Te network. This innovation presents a promising technique for fabricating high-performance Te TFTs.

## Figures and Tables

**Table 1:** Representative transfer parameters of Te TFTs under various conditions.

Thickness	µ_FE_ (cm^2^/Vs)	S.S. (V/dec)	V_on_ (V)	I_ON_/I_OFF_
5 nm	0.58	2.70	0.03	2.24 × 10^4^
7 nm	2.68	10.46	−0.88	6.36 × 10^2^
7 nm Pristine	2.16	14.03	−3.11	2.95 × 10^2^
7 nm with In Capping	33.54	12.52	−2.21	1.05 × 10^3^
9 nm	3.92	22.84	9.42	4.86 × 10^1^

## Data Availability

The data presented in this study are available upon request from the corresponding author.

## Supplementary Materials

The following supporting information can be downloaded at: https://www.mdpi.com/article/10.3390/nano15060418/s1. Figure S1: OM image of a Te thin film according to annealing temperature. Table S1: Summary of Te-related TFTs.
