# Supplementary material for: High-Mobility Tellurium Thin-Film Transistor: Oxygen Scavenger Effect Induced by a Metal-Capping Layer
Source: Nanomaterials (Basel). 2025 Mar 8;15(6):418. doi: 10.3390/nano15060418 (PMC11945103; doi:10.3390/nano15060418)
Supplement: Supplementary file 1 [file nanomaterials-15-00418-s001.zip › nanomaterials-3483571-supplementary.pdf]

nanomaterials

MDPI

Article

High-Mobility Tellurium Thin-Film Transistor: Oxygen Scavenger Effect Induced by a Metal-Capping Layer

Seung-Min Lee[1†], Seong Cheol Jang[2†], Ji-Min Park[2], Jaewon Park[2], Nayoung Choi[3], Kwun-Bum Chung[3], Jung Woo Lee[4], and Hyun-Suk Kim[2,*]

[1] Department of Materials Science and Engineering, Chungnam National University, Daejeon (34134), Republic of Korea;
[2] Department of Energy and Materials Engineering, Dongguk University, Seoul (04620), Republic of Korea;
[3] Department of Physics, Dongguk University, Seoul (04620), Republic of Korea;
[4] School of Materials Science and Engineering, Pusan National University, Busan (46241), Republic of Korea.;

* Correspondence: khs3297@dongguk.edu
† These authors contributed equally to this work.

Academic Editor: Firstname Lastname

Received: date
Revised: date
Accepted: date
Published: date

Citation: To be added by editorial staff during production.

Copyright: © 2025 by the authors. Submitted for possible open access publication under the terms and conditions of the Creative Commons Attribution (CC BY) license (https://creativecommons.org/licenses/by/4.0/).

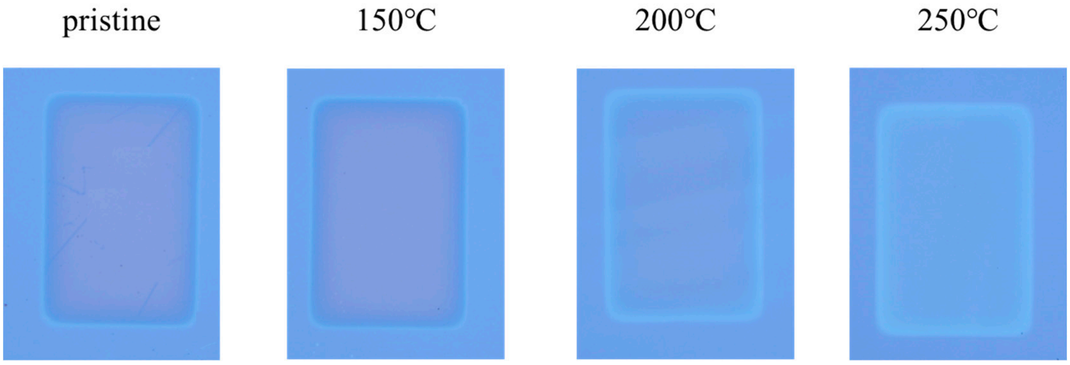

Figure S1. OM images of a Te thin film according to the annealing temperature.

Table S1. Summary of Te-related TFTs.

Ref.	Process	Fabrication Temp. (°C)	Mobility (cm^2/Vs)	On/off	SS (mV/dec)	V_{th} (V)	
S1[1]	Evap.	-80	~ 35	~ 10^4	108	-	
S2[2]	Sputter	25	12.05	-	434	0.09	Strain modulation
S3[3]	Sputter	RT	-	~ 10^5	30	-	
S4[4]	Evap.	225	15	10^6-10^7	-	-	Selenium-alloyed tellurium oxide
S5[5]	MBE	120	707	10^3	-	10	Needle-like structures
S6[6]	PLD	RT	3	3 x 10^5	2100	-	Se−Te Alloying
S7[7]	Evap.	-10	21.1	10^4	880	1.0	Facile polyethyleneimine (PEI) surface treatment
S8[8]	Sputter	RT	30.9	5.8 x 10^5	650	1.4	Alumina encapsulation
S9[9]	Sputter	RT	38.0	3.8 x 10^5	670	2.8	Partial oxide
This work	Sputter	RT	2.68	6.36 x 10^2	1046	-0.88	
This work	Sputter	RT	33.54	1.05 x 10^3	1252	-3.11	In-capping layer

References

1. Zhao, C.; Tan, C.; Lien, D.-H.; Song, X.; Amani, M.; Hettick, M.; Nyein, H.Y.Y.; Yuan, Z.; Li, L.; Scott, M.C. Evaporated tellurium thin films for p-type field-effect transistors and circuits. *Nature nanotechnology* **2020**, *15*, 53-58.
2. Oh, J.-S.; Kim, T.I.; Kwon, H.-I.; Park, I.-J. Strain modulation effects on two-dimensional tellurium for advanced p-type transistor applications. *Applied Surface Science* **2024**, *651*, 159288.
3. Wang, X.; Li, H. A steep-slope tellurium transistor with a native voltage amplifying threshold switch. *Applied Physics Letters* **2022**, *120*.
4. Liu, A.; Kim, Y.-S.; Kim, M.G.; Reo, Y.; Zou, T.; Choi, T.; Bai, S.; Zhu, H.; Noh, Y.-Y. Selenium alloyed tellurium oxide for amorphous p-channel transistors. *Nature* **2024**, 1-3.
5. Zhou, G.; Addou, R.; Wang, Q.; Honari, S.; Cormier, C.R.; Cheng, L.; Yue, R.; Smyth, C.M.; Laturia, A.; Kim, J. High-mobility helical tellurium field-effect transistors enabled by transfer-free, low-temperature direct growth. *Advanced Materials* **2018**, *30*, 1803109.
6. Choi, K.; Nam, S.; Kim, Y.-H.; Oh, H.; Kim, I.; Lee, K.; Cho, S.H. Promotion of Processability in a p-Type Thin-Film Transistor Using a Se–Te Alloying Channel Layer. *ACS Applied Materials & Interfaces* **2024**, *16*, 23459-23466.
7. Kim, G.H.; Kang, S.-H.; Lee, J.M.; Son, M.; Lee, J.; Lee, H.; Chung, I.; Kim, J.; Kim, Y.-H.; Ahn, K. Room temperature-grown highly oriented p-type nanocrystalline tellurium thin-films transistors for large-scale CMOS circuits. *Applied Surface Science* **2023**, *636*, 157801.
8. Kim, T.; Choi, C.H.; Byeon, P.; Lee, M.; Song, A.; Chung, K.-B.; Han, S.; Chung, S.-Y.; Park, K.-S.; Jeong, J.K. Growth of high-quality semiconducting tellurium films for high-performance p-channel field-effect transistors with wafer-scale uniformity. *npj 2D Materials and Applications* **2022**, *6*, 4.
9. Kim, T.; Choi, C.H.; Kim, S.E.; Kim, J.-K.; Jang, J.; Choi, S.; Noh, J.; Park, K.-S.; Kim, J.; Yoon, S. High-performance hexagonal tellurium thin-film transistor using tellurium oxide as a crystallization retarder. *IEEE Electron Device Letters* **2022**, *44*, 269-272.
